# 3,3′-Diindolylmethane (DIM) and its ring-substituted halogenated analogs (ring-DIMs) induce differential mechanisms of survival and death in androgen-dependent and –independent prostate cancer cells

**DOI:** 10.18632/genesandcancer.60

**Published:** 2015-05

**Authors:** Alexander A. Goldberg, Hossam Draz, Diana Montes-Grajales, Jesus Olivero-Verbél, Stephen H. Safe, J. Thomas Sanderson

**Affiliations:** ^1^ INRS-Institut Armand-Frappier, Laval, Québec, Canada; ^2^ Critical Care Division and Meakins-Christie Laboratories, Faculty of Medicine, McGill University, Montreal, Quebec H3A 1A1, Canada; ^3^ Department of Biochemistry, National Research Centre, Dokki, Cairo, Egypt; ^4^ Environmental and Computational Chemistry Group, University of Cartagena, Colombia; ^5^ Veterinary Physiology and Pharmacology, Texas A&M University, College Station, TX, United States

**Keywords:** prostate cancer, LNCaP, C42B, DU145, mitochondrial function

## Abstract

We recently reported that novel ring-substituted analogs of 3,3′-diindolylmethane (ring-DIMs) induce apoptosis and necrosis in androgen-dependent and –independent prostate cancer cells. In this paper, we have focused on the mechanism(s) associated with ring-DIM-mediated cell death, and on identifying the specific intracellular target(s) of these compounds. The 4,4′- and 7,7′-dichloroDIMs and 4,4′- and 7,7′-dibromoDIMs induced the death of LNCaP, C42B and DU145 prostate cancer cells, but not that of immortalized normal human prostate epithelial (RWPE-1) cells. Ring-DIMs caused the early loss of mitochondrial membrane potential (MMP) and decreased mitochondrial ATP generation in prostate cancer cells. Cyclosporin A, an inhibitor of the mitochondrial permeability transition pore, inhibited ring-DIM-mediated cell death, and salubrinal, an inhibitor of ER stress, inhibited cell death mediated only by 4,4′-dihaloDIMs. We found that although salubrinal did not inhibit the onset of ER stress, it prevented 4,4′-dibromoDIM mediated loss of MMP. Salubrinal potentiated cell death in response to 7,7′-dihaloDIMs and DIM, and this effect concurred with increased loss of MMP. Using *in silico* 3-D docking affinity analysis, we identified Ca_2+_/calmodulin-dependent kinase II (CaMKII) as a potential direct target for the most toxic ring-DIM, 4,4′-dibromoDIM. An inhibitor of CaMKII, KN93, but not its inactive analog KN92, abrogated cell death mediated by 4,4′-dibromoDIM. The ring-DIMs induced ER stress and autophagy, but these processes were not necessary for ring-DIM-mediated cell death. Inhibition of autophagy with bafilomycin A1, 3-methyladenine or by LC3B gene silencing sensitized LNCaP and C42B, but not ATG5-deficient DU145 cells to ring-DIM- and DIM-mediated cell death. We propose that autophagy induced by the ring-DIMs and DIM has a cytoprotective function in prostate cancer cells.

## INTRODUCTION

Prostate cancer accounts for almost one third of all cancer deaths in the United States and is the second highest cause of cancer-related death in males [1]. Most prostate tumours are initially androgen-dependent (AD). However, a large contingent will progress to an aggressive androgen-independent (AI) form, which are more drug-resistant and lead to increased morbidity and mortality among patients. Currently, prostate cancer is treated with a combination of radiotherapy, chemical castration, androgen receptor (AR) antagonists (hydroxyflutamide, bicalutamide), or inhibitors of steroidogenesis (abiraterone). Patients treated with hydroxyflutamide or bicalutamide often suffer from severe side-effects as a result of the anti-androgenic therapy [2, 3], necessitating the search for novel chemotherapeutic agents with fewer deleterious effects. Moreover, it is imperative to search for novel therapeutic targets which may aid the development of a new generation of drugs effective in the elimination of AI prostate tumours.

3,3′-Diindolylmethane (DIM) is a natural small molecule produced in the stomach after ingestion of vegetables of the *Brassica* family containing high amounts of indole-3-carbinol (I3C). I3C is converted via acid-catalyzed reactions in the stomach to various condensation products, of which DIM is considered its most biologically active metabolite [4, 5]. DIM has been studied extensively as an anticancer agent due to its ability to inhibit the growth of a multitude of cancer cell types *in vitro* and *in vivo* [6, 7] and has produced positive responses in clinical trials for the treatment of prostate cancer when applied in an absorption-enhanced formulation [8]. DIM affects a number of distinct yet overlapping pathways, leading to the inhibition of cancer cell proliferation. For example, DIM down-regulates AR transcriptional activity, thereby reducing AR-mediated gene expression [9-12]. DIM also inhibits pro-survival cell signaling pathways such as phosphatidylinositide 3-kinase (PI3K), Akt, mammalian target of rapamycin (mTOR) and c-Met, and also activates pro-apoptotic pathways such as Hippo and glycogen synthase kinase 3-beta (GSK-3β), resulting in inhibition of cancer cell proliferation [13-19]. DIM activates the pro-apoptotic proteins Fas, FasL and death receptor 5 (DR5), leading to caspase-dependent apoptosis [7, 9, 17, 20]. DIM also increases the intracellular flux of calcium ions, resulting in the induction of endoplasmic reticulum (ER) stress genes [21-23], in addition to decreasing mitochondrial function through inhibition of ATP synthase [24-26], which in turn induces AMP-activated protein kinase-(AMPK)-dependent autophagy [27]. DIM also exerts effects on DNA methyltranferases, resulting in modified methylation patterns of genes involved in inflammation, cell signaling, cell motility and apoptosis [28]. However, the specific molecular targets that directly interact with DIM to cause ER stress, mitochondrial dysfunction, autophagy, and ultimately cell death have yet to be discerned.

We have previously shown that several halogenated analogs of DIM, termed ring-DIMs, act as anti-androgenic compounds that inhibit AD proliferation of LNCaP human prostate cancer cells and induce apoptosis and necrosis of AD as well as AI prostate cancer cells with greater potencies than DIM [11, 17]. Cell death induced only by the most potent ring-DIM, 4,4′-Br_**2**_DIM, was partially dependent on activation of caspase-3, which occurred concomitant with increases in Fas, FasL, DR4 and DR5 expression. The objective of the present study was to determine the early events that ultimately result in cell death induced by 4,4′- and 7,7′-dibromo- and dichloro-substituted ring-DIMs and DIM by determining their concentration- and time-dependent effects on mitochondrial stability, ER stress and autophagy. We also performed an *in silico* docking affinity analysis to identify proteins that could potentially interact with ring-DIMs and DIM.

## RESULTS

### Ring-DIMs kill LNCaP, C42B and DU145 prostate cancer cells, but not RWPE-1 immortalized normal prostate epithelial cells

**Figure 1:**
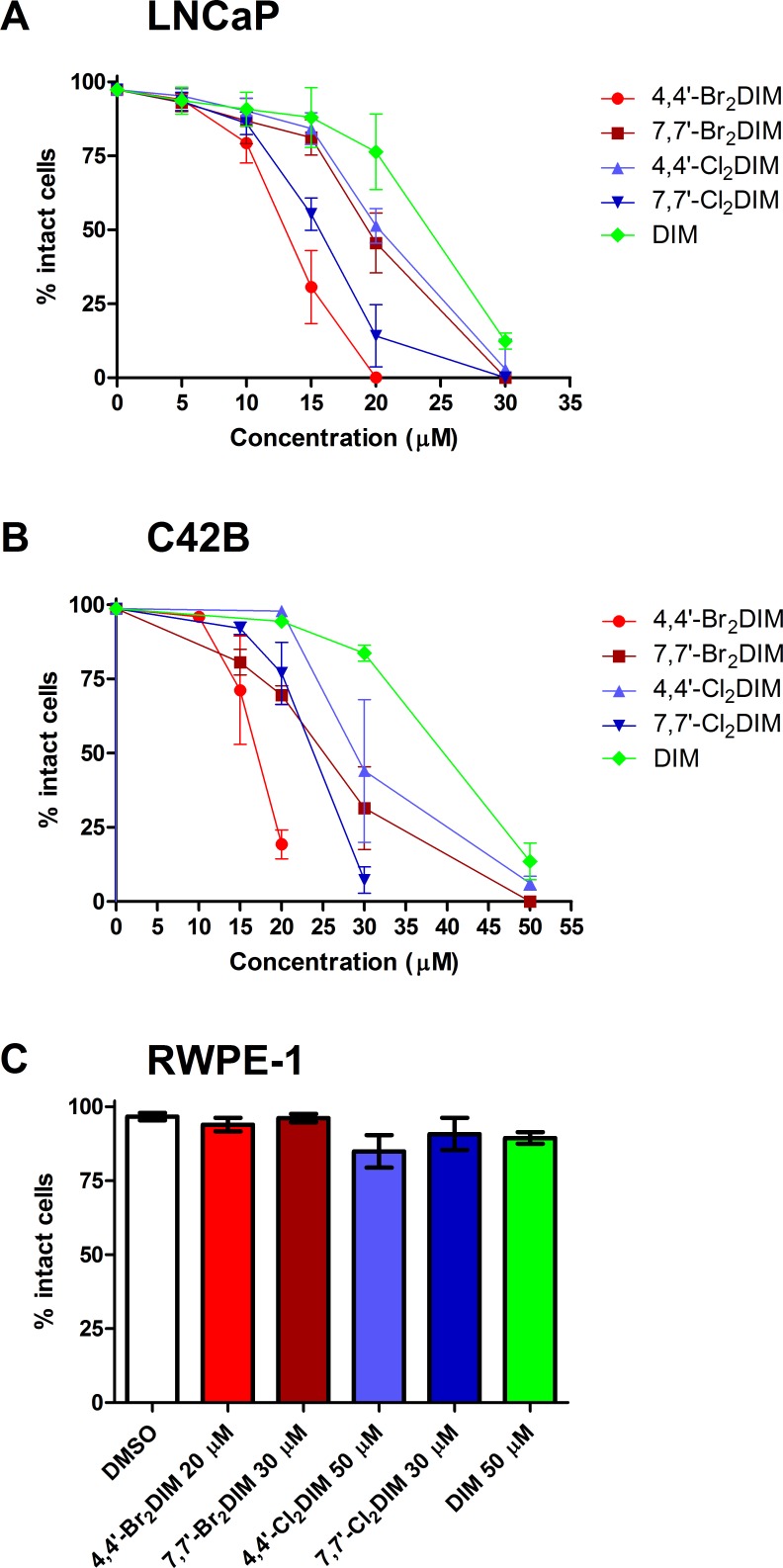
Ring-DIMs kill LNCaP and C42B prostate cancer cells, but not RPWE-1 immortalized normal prostate epithelial cells The percentage of intact LNCaP **(A)** and C42B **(B)** cells that do not have fragmented nuclei, condensed chromatin or propidium iodide staining was determined after a 24 hour exposure to increasing concentrations of 4,4′-Br_2_DIM, 7,7′-Br_2_DIM, 4,4′-Cl_2_DIM, 7,7′-Cl_2_DIM or DIM. **(C)** Percentage of intact RWPE-1 cells after a 48 hour exposure to the ring-DIMs or DIM.

We tested the ability of the ring-DIMs to kill prostate cancer cells that express a DHT-responsive AR (LNCaP), a constitutively active AR (C42B) and cells lacking AR (DU145). In LNCaP and C42B cells, 4,4′-Br_**2**_DIM (IC_**50**_ = 13.1 μM, 16.7 μM, respectively), 4,4′-Cl_**2**_DIM (IC_**50**_ = 20.2 μM, 29.3), 7,7′-Br_**2**_DIM (IC_**50**_ = 19.5 μM, 25.3 μM) and 7,7′-Cl_**2**_DIM (IC_**50**_ = 15.8 μM, 25.7 μM) were all significantly more potent at killing cells than DIM (IC_**50**_ = 23.3, 46.1 μM), (Fig. 1A, B). The most cytotoxic ring-DIM, 4,4′-Br_**2**_DIM, killed AR-negative DU145 cells with the same potency as C42B cells with an IC_**50**_ of 20 μM (Supplementary Fig. S1A). At concentrations that were toxic to the prostate cancer cells neither the ring-DIMs nor DIM induced cell death in RWPE-1 cells (Fig. 1C).

### Mitochondrial dysfunction and ER stress are early events in ring-DIM induced cell death

**Figure 2:**
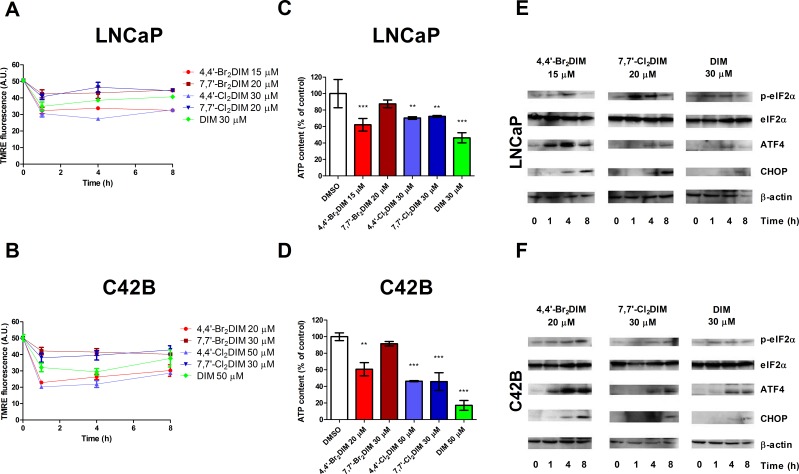
Mitochondrial dysfunction and ER stress are early events in ring-DIM- and DIM-mediated prostate cancer cell death Tetramethylrhodamine ethyl ester (TMRE) fluorescence of LNCaP **(A)** and C42B **(B)** cells after 0, 1, 4, and 8 hrs of exposure to 4,4′-Br_2_DIM, 7,7′-Br_2_DIM, 4,4′-Cl_2_DIM, 7,7′-Cl_2_DIM or DIM. Relative mitochondrial ATP levels of LNCaP **(C)** and C42B **(D)** cells treated with 5 mM 2-deoxy-D-glucose after a 1 h exposure to 4,4′-Br_2_DIM, 7,7′-Br_2_DIM, 4,4′-Cl_2_DIM, 7,7′-Cl_2_DIM or DIM. Phosphorylation of eIF2α, and levels of ER stress proteins were assayed by immunoblot of LNCaP **(E)** and C42B **(F)** cells after 0, 1, 4, and 8 hrs of exposure to 4,4′-Br_2_DIM, 7,7′-Cl_2_DIM or DIM.

To further investigate the mechanism of ring-DIM-induced toxicity, we looked at MMP, mitochondrial ATP generation and ER stress in response to ring-DIM exposure. After only 1 hour of exposure to the 4,4′-dihaloDIMs, MMP decreased by 36-40% in LNCaP and 54-60% in C42B cells, whereas MMP decreased by only 30% in LNCaP and 36 % in C42B cells after a 1 hour treatment with DIM. The 7,7′-dihaloDIMs (Fig. 2A, B) decreased MMP by only 15-18% in LNCaP and 16-24% in C42B cells. The observed decreases in MMP were sustained for at least 8 hours. DIM and all ring-DIMs, except 7,7′-Br_**2**_DIM, significantly decreased mitochondrial ATP generation in both cell lines by up to 80% (Fig. 2C, D). In DU145 cells treated with 4,4-Br_**2**_DIM, we observed decreases in MMP by 63% and ATP generation by 45% compared to controls (Supplementary Fig. S1B, C).

Treatment of LNCaP and C42B cells with 4,4′-Br_**2**_DIM, 7,7′-Cl_**2**_DIM or DIM also caused a significant increase in the levels of expression of ER stress-related proteins CHOP and ATF4 and the phosporylation of eIF2α between 1 and 8 hours of exposure (Fig. 2E, F). These results were also observed in DU145 cells using 4,4-Br_**2**_DIM, which also increased ATF4 and CHOP levels after treatment for 1 to 8 hours (Supplementary Fig. S1D).

### Ring-DIM-induced cell death is dependent on mitochondrial dysfunction

**Figure 3:**
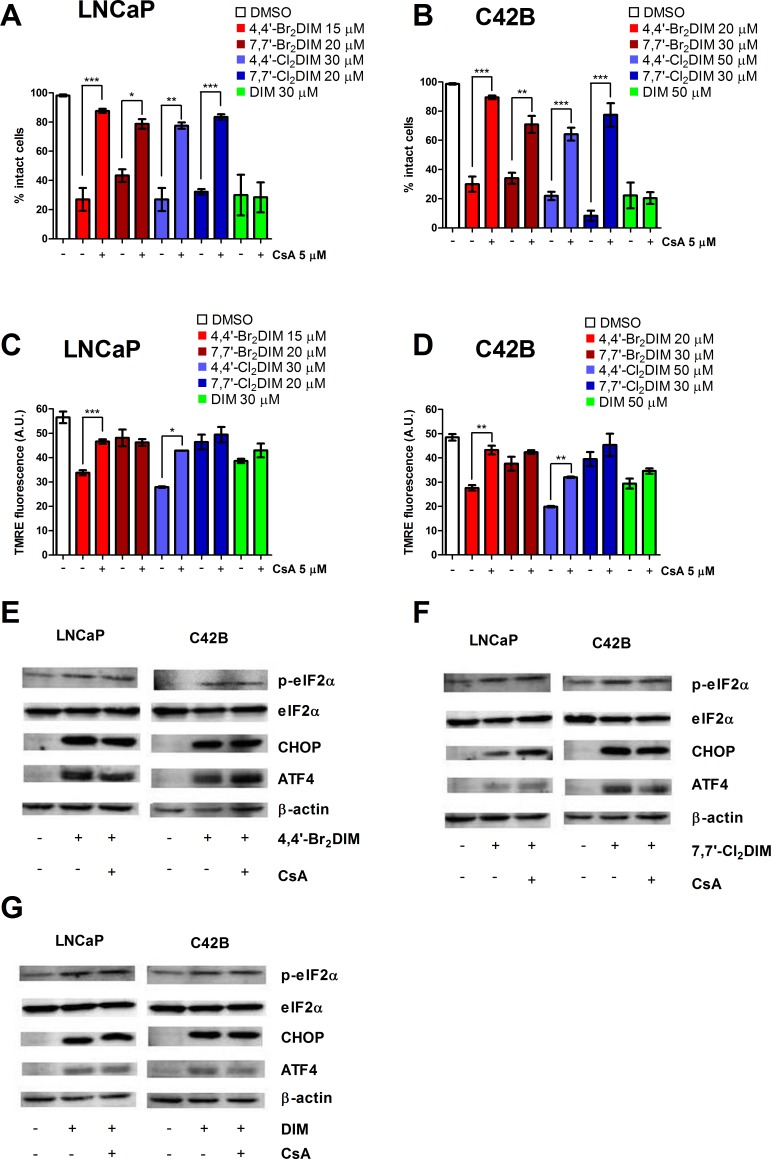
Ring-DIM mediated cell death is dependent on the mitochondrial permeability transition pore (mPTP) Percentage of intact LNCaP **(A)** and C42B **(B)** cells after a 24 h exposure to 4,4′-Br_2_DIM, 7,7′-Br_2_DIM, 4,4′-Cl_2_DIM, 7,7′-Cl_2_DIM, or DIM with or without a 4 h pre-treatment with cyclosporin A (CsA). TMRE fluorescence of LNCaP **(C)** or C42B **(D)** cells after a 4 h exposure to 4,4′-Br_2_DIM, 7,7′-Br_2_DIM, 4,4′-Cl_2_DIM, 7,7′-Cl_2_DIM, or DIM with or without a 4 h pre-treatment with cyclosporin A (CsA). Phosphorylation of eIF2α, and levels of ER stress proteins in LNCaP and C42B cell extracts after a 24 hour exposure to 4,4′-Br_2_DIM **(E)**, 7,7′-Cl_2_DIM **(F)** or DIM **(G)** with or without a 4 hour pre-treatment with CsA.

We next assessed whether CsA, an inhibitor of the mitochondrial permeability transition pore, could abrogate the toxicity of cells exposed to the ring-DIMs or DIM. We found that pre-treatment with 5 μM of CsA prevented ring-DIM-mediated loss of cell viability, but did not affect DIM-induced death of LNCaP or C42B cells (Fig. 3A, B). In LNCaP and C42B cells, CsA prevented the loss of MMP caused by the 4,4′-dihaloDIMs and, to a lesser extent, DIM, but had no effect on the loss of MMP mediated by the 7,7′-dihaloDIMs (Fig. 3C, D). We confirmed that CsA also prevented 4,4′-Br_**2**_DIM-mediated cell death and loss of MMP in AI DU145 cells (Supplementary Fig. S1E and B). However, CsA had no effect on the phosphorylation status of eIF2α or the expression levels of CHOP and ATF4 in LNCaP or C42B cells treated with either of the three compounds (Fig. 3E-G); this was confirmed using only 4,4′- Br_**2**_DIM in DU145 cells (Supplementary Fig. S1F). Treatment with CsA alone did not affect cell viability or eIF2α phosphorylation in LNCaP, C42B or DU145 cells (Supplementary Fig. S2A, B); nor did it significantly affect MMP (Supplementary Fig. S2C).

### Salubrinal inhibits cell death and loss of MMP but not ER stress mediated by 4,4′-Br_2_DIM

**Figure 4:**
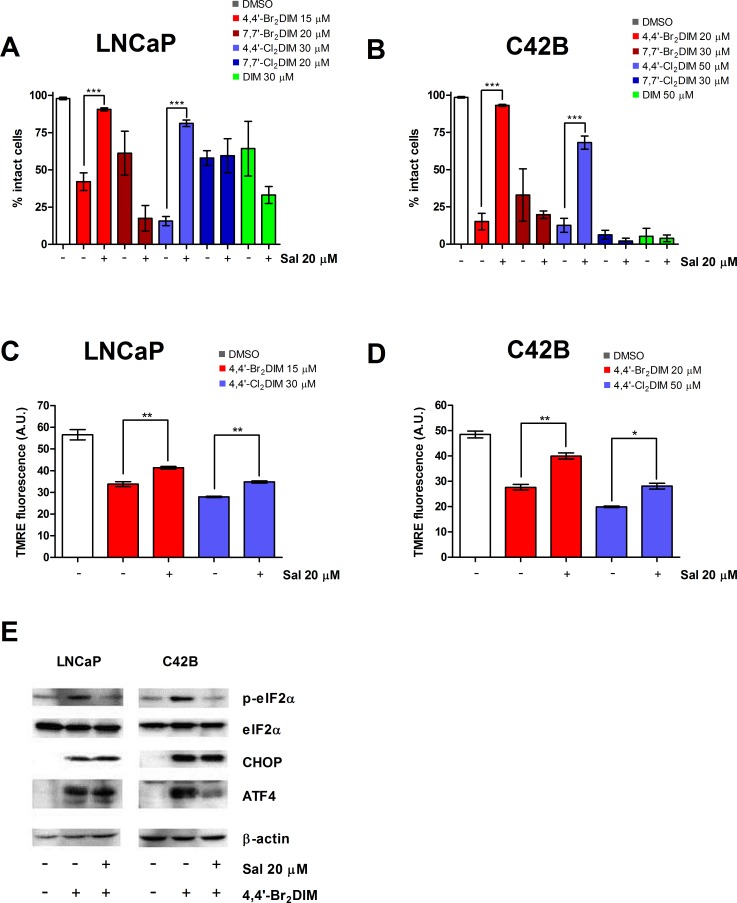
Salubrinal modulates mitochondrial activity in prostate cancer cells treated with 4,4′-dihaloDIMs The percentage of intact LNCaP **(A)** and C42B **(B)** cells was calculated after a 24 hour exposure to toxic concentrations of 4,4′-Br_2_DIM, 7,7′-Br_2_DIM, 4,4′-Cl_2_DIM, 7,7′-Cl_2_DIM, DIM with or without a 4 h pre-treatment with salubrinal. TMRE fluorescence of LNCaP **(C)** and C42B **(D)** cells after a 4 hour exposure to 4,4′-Br_2_DIM or 4,4′-Cl_2_DIM with or without a 4 h pre-treatment with salubrinal. **(E)** Phosphorylation of eIF2α, and levels of ER stress proteins after a 24 hour exposure to 4,4′-Br_2_DIM with or without a 4 hour pre-treatment with salubrinal.

Salubrinal is an inhibitor of ER stress that blocks dephosphorylation of eIF2α, and we investigated the effects of this compound on the cytotoxicity of the ring-DIMs and DIM. Pre-treatment of LNCaP and C42B cells with 20 μM of salubrinal inhibited cell death caused by 4,4′-dihaloDIMs, but not 7,7′-dihaloDIMs or DIM (Fig 4A, B); in DU145 cells salubrinal pre-treatment also completely prevented 4,4′-Br_**2**_DIM-induced cell death (Supplementary Fig. S1E).

In cells exposed to 4,4′-Br_**2**_DIM or 4,4′-Cl_**2**_DIM, pre-treatment with salubrinal attenuated the 4,4′-dihaloDIM-mediated decrease in MMP (Fig 4C, D) (Supplementary Fig. S1B). Salubrinal pre-treatment alone did not significantly affect MMP in LNCaP, C42B or DU145 cells (Supplementary Fig. S2C). Using the most toxic of the 4,4′-dihaloDIMs as prototype, the 4,4′-Br_**2**_DIM-mediated increase in phosphorylation of eIF2α was inhibited by pre-treatment with salubrinal in all three cell lines (Fig. 4E and Supplementary Fig. S1G). However, in LNCaP and C42B cells, pre-treatment with salubrinal did not consistently abrogate the 4,4′-Br_**2**_DIM-induced levels of CHOP or ATF4 (Fig. 4E), and in DU145 cells salubrinal only partially reduced the 4,4′-Br_**2**_DIM-induced expression of these markers of ER stress (Supplementary Figure S1G). Salubrinal alone did not affect cell viability or eIF2α phosphorylation in either of the three cell lines (Supplementary Figure S2A, B).

### Salubrinal potentiates 7,7′-dihaloDIM- and DIM-mediated toxicity via loss of MMP

**Figure 5:**
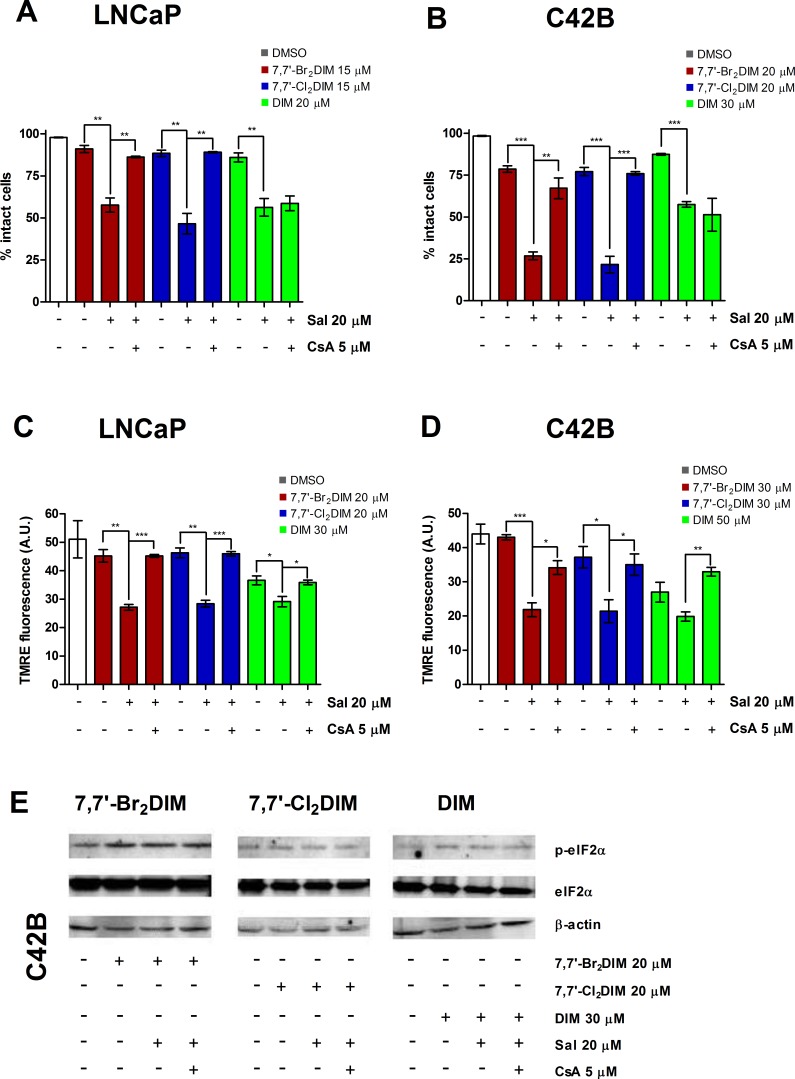
Cyclosporin A (CsA) abrogates salubrinal-mediated sensitization of prostate cancer cells to mitochondrial dysfunction using sub-toxic concentrations of 7,7′-dihaloDIMs or DIM The percentage of intact LNCaP **(A)** and C42B **(B)** cells pre-treated for 4 hours with salubrinal, salubrinal and CsA, or 0.3% DMSO, followed by a 24 hour exposure to mildly toxic concentrations of 7,7′-Br_2_DIM, 7,7′-Cl_2_DIM or DIM. TMRE fluorescence of LNCaP **(C)** and C42B **(D)** cells pre-treated for 4 hours with salubrinal and cyclosporin A (CsA), or 0.3% DMSO followed by a 4 hour exposure to 7,7′-Br_2_DIM or 7,7′-Cl_2_DIM. **(E)** Phosphorylation of eIF2α in C42B cell extracts after a 24 hour exposure to mildly toxic concentrations of 7,7′-Br_2_DIM, 7,7′-Cl_2_DIM or DIM with or without a 4 hour pre-treatment with salubrinal, salubrinal and CsA, or 0.3 % DMSO.

Next, we asked if salubrinal could sensitize prostate cancer cells to cell death induced by the 7,7′-dihaloDIMs and DIM. Pre-treatment of LNCaP and C42B cells with salubrinal enhanced the loss of cell viability caused by concentrations of the 7,7′-dihaloDIMs or DIM that are otherwise sub-toxic (defined as all cells being intact and not visibly Hoechst- or PI-stained) in cells that are exposed to the 7,7′-dihaloDIMs alone. Loss of viability mediated by co-treatment of LNCaP or C42B cells with salubrinal and sub-toxic concentrations of 7,7′-dihaloDIMs was attenuated by pre-treatment with CsA (Fig. 5A, B). Consistent with the lack of abrogation of the cytotoxicity of DIM by CsA alone (Fig 3A, B) CsA was also incapable of abrogating the cytotoxicity of DIM that was potentiated by pre-treatment with salubrinal (Fig. 5A, B)

LNCaP and C42B cells pre-treated with salubrinal and treated with either 7,7′-Br_**2**_DIM, 7,7′-Cl_**2**_DIM or DIM, exhibited a sharp decrease in MMP after 4 hours (Fig. 5C, D). Combined pre-treatment of cells with salubrinal and CsA partially or fully restored the loss of MMP caused by 7,7′-Cl_**2**_DIM, 7,7′-Br_**2**_DIM and DIM (Fig 5C, D). Neither salubrinal nor combined salubrinal and CsA pre-treatments affected eIF2α phosphorylation in response to sub-toxic concentrations of 7,7′-dihaloDIMs or DIM as determined in C42B cells (Fig. 5E).

### Ring-DIMs and DIM induce protective autophagy in prostate cancer cells

**Figure 6:**
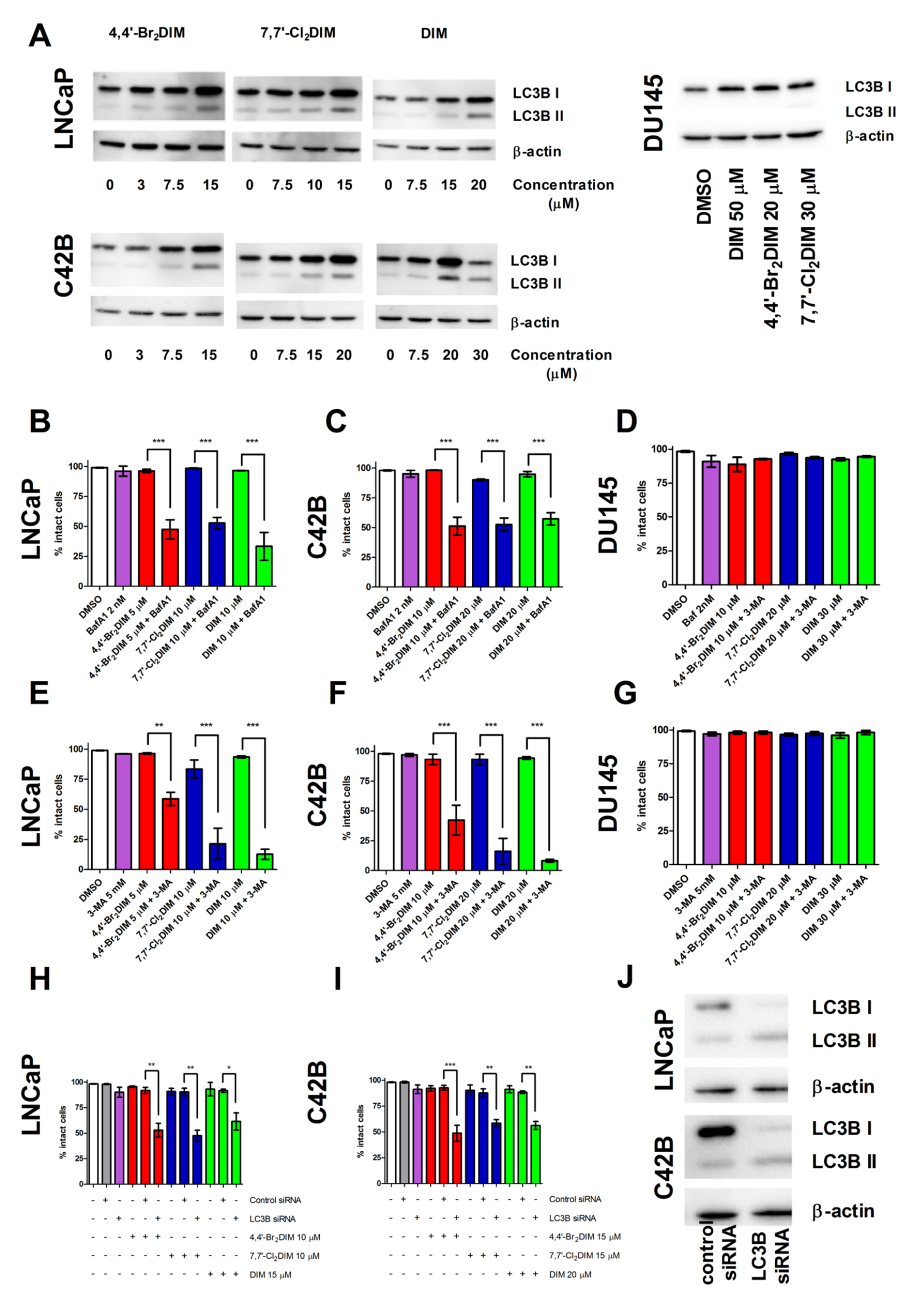
Ring-DIMs and DIM induce protective autophagy **(A)** Levels of LC3B I and II protein in LNCaP, C42B and DU145 cells after a 24 hr exposure to 4,4′-Br_2_DIM, 7,7′-Cl_2_DIM or DIM. Percentage of intact LNCaP **(B, E)**,C42B **(C, F)** and DU145 **(D, G)** cells after a 24 h exposure to 4,4′-Br_2_DIM, 7,7′-Cl_2_DIM or DIM with or without a 4 hour pre-treatment with bafilomycin A1 (BafA1) or 3-methyladenine (3-MA). Percentage of intact LNCaP **(H)** and C42B **(I)** cells after a 24 hour exposure to 4,4′-Br_2_DIM, 7,7′-Cl_2_DIM or DIM with or without a 24 hour pre-treatment with LC3B siRNA. **(J)** Protein levels of LC3B I and II in LNCaP and C42B cells after treatment with or without LC3B siRNA.

We investigated the potential of DIM and the ring-DIMs to induce autophagy in prostate cancer cells. A dose-dependent increase in LC3B conversion was observed in LNCaP and C42B cells, but not in autophagy-deficient DU145 cells, exposed to sub-toxic concentrations of 4,4′-Br_**2**_DIM, 7,7′-Cl_**2**_DIM or DIM for 24 hours (Fig. 6A). Pre-treatment of cells with autophagy inhibitors bafilomycin A1 (Fig. 6B-D) or 3-MA (Fig. 6E-G) sensitized LNCaP and C42B cells, but not DU145 cells, to sub-toxic concentrations of 4,4′-Br_**2**_DIM, 7,7′-Cl_**2**_DIM or DIM. We next silenced the LC3B gene in LNCaP and C42B cells using siRNA and then exposed them to sub-toxic concentrations of 4,4′-Br_**2**_DIM, 7,7′-Cl_**2**_DIM or DIM. We observed significant decreases in viability of cells treated with the LC3B-selective siRNA, but not control siRNA, with each of the three compounds (Fig. 6H, I). We confirmed the effectiveness of the LC3B-selective siRNA in decreasing the expression of LC3B in LNCaP and C42B cells to almost undetectable levels relative to control siRNA (Fig. 6J).

### *In silico* identification of protein targets for ring-DIMs and DIM

To identify potential molecular targets for the ring-DIMs and DIM, we performed *in silico* 3-D affinity docking studies of 61 proteins with known 3-D crystal structures and which are involved in cell survival or death and mitochondrial function, to determine possible high-affinity interactions with the 4,4′-dihaloDIMs, 7,7′-dihaloDIMs and DIM. We identified CaMKII subunits alpha, beta and gamma, but not delta as possible high-affinity targets common to all five compounds (Table 1). Each compound had *in silico* docking affinities for each of the subunits with free energy values less than −8.5 kcal/mol, with a value lower than −8.0 kcal/mol considered a high-affinity interaction.

**Table 1:** Docking affinities (kcal/mol) of DIM and its derivatives for the four isoforms of the calmodulin-dependent kinase II protein involved in mitochondrial metabolism and signaling pathways

Short name	Pathway	PDB ID	Docking affinity (kcal/mol)				
			4,4′-dibromo DIM	4,4′dichloroDIM	7,7′-dibromo DIM	7,7′-dichloro DIM	diindolylmethane (DIM)
CaMK-II subunit α	Signal transduction	2VZ6/Q9UQM7	−8.8±0.0	−9.0±0.0	−8.8±0.1	−8.9±0.1	−9.2±0.0
CaMK-II subunit β	Signal transduction	3BHH/Q13554	−9.5±0.0	−9.5±0.0	−9.0±0.0	−8.9±0.1	−9.1±0.0
CaMK-II subunit γ	Signal transduction	2V7O/Q13555	−8.9±0.1	−9.1±0.0	−9.1±0.7	−8.6±0.8	−9.2±0.1
CaMK-II subunit δ	Signal transduction	2WEL/Q13557	−7.4±0.0	−7.6±0.0	−7.4±0.1	−7.7±0.1	−7.5±0.0

### KN93 abrogates cell death induced by 4,4′-Br_2_DIM

**Figure 7:**
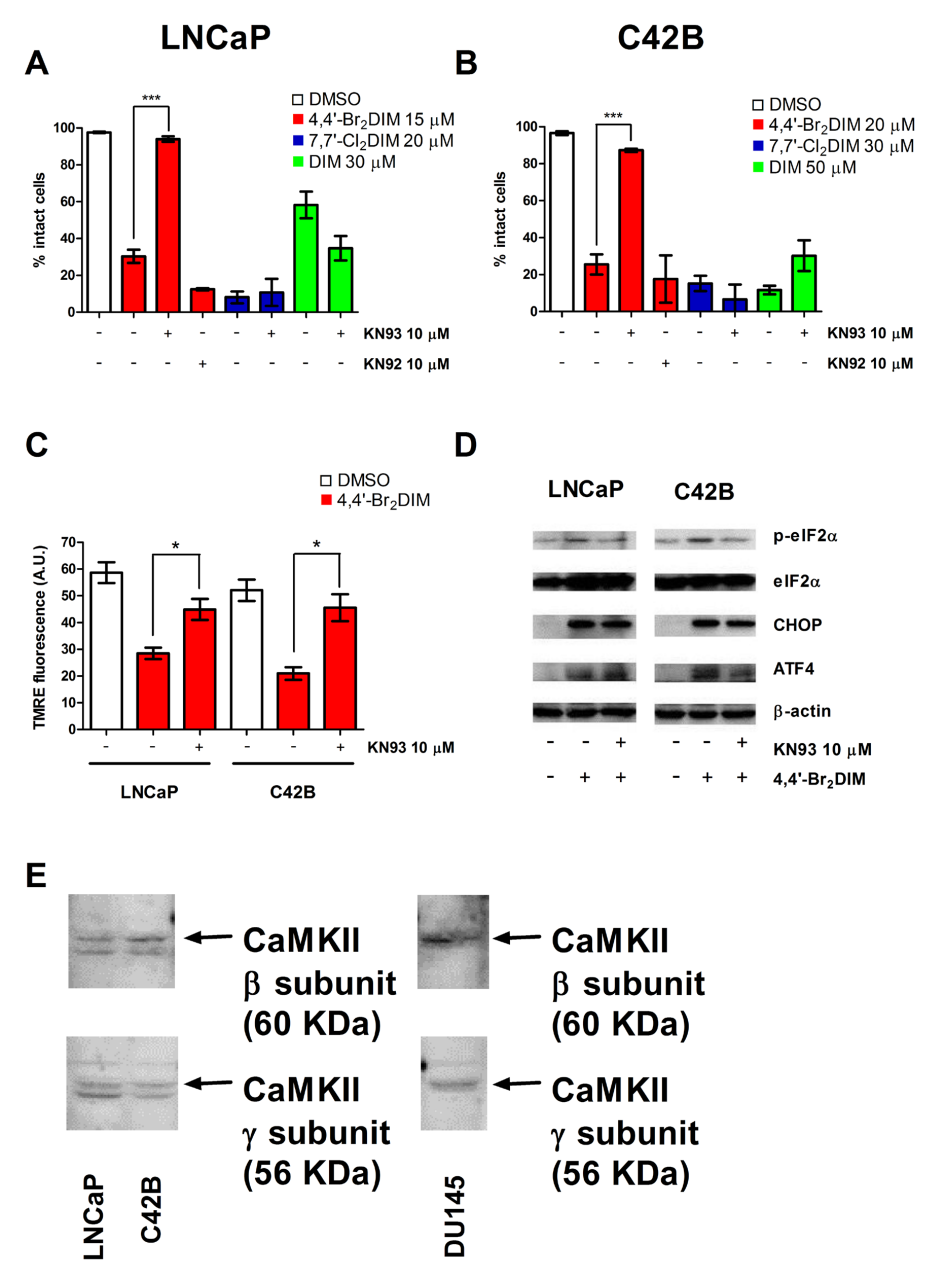
KN93 abrogates 4,4′-Br2DIM mediated cell death Percentage of intact LNCaP **(A)** and C42B **(B)** cells after a 24 hour exposure to 4,4′-Br_2_DIM, 7,7′-Cl_2_DIM or DIM with or without a 4 hour pre-treatment with KN92 or KN93. **(C)** TMRE fluorescence of LNCaP and C42B cells after a 4 hour exposure to 4,4′-Br_2_DIM with or without a 4 hour pre-treatment with KN93. **(D)** Phosphorylation of eIF2α, and levels of ER stress proteins in LNCaP and C42B cells after a 24 hour exposure to 4,4′-Br_2_DIM with or without a 4 hour pre-treatment with KN93. **(E)** Protein levels of Ca_2+_/calmodulin-dependent protein kinase (CaMKII) beta and gamma subunits in LNCaP, C42B and DU145 cells.

The involvement of the various CaMKII subunits in DIM or ring-DIM-mediated toxicity in prostate cancer cells was assessed using a selective CaMKII inhibitor, KN93. In LNCaP, C42B and DU145 cells, a 4 hour pre-treatment with KN93 resulted in a marked reduction of cell death caused by 4,4′-Br_**2**_DIM, but not 7,7′-Cl_**2**_DIM or DIM (Fig. 7A-B, Supplementary Fig. S1E). Additionally, KN92, an inactive derivative of KN93, did not abrogate cell death induced by 4,4′-Br_**2**_DIM (Fig. 7A-B, Supplementary Fig. S1E). Pre-treatment with KN93 restored the loss of MMP caused by 4,4′-Br_**2**_DIM in all three prostate cancer cell lines (Fig. 7C, Supplementary Fig. S1B). Pre-treatment of LNCaP and C42B cells with KN93 reduced 4,4′-Br_**2**_DIM-mediated eIF2α phosphorylation, but it did not affect 4,4′-Br_**2**_DIM-mediated increases in CHOP and ATF4 levels (Fig. 7D). In DU-145 cells, pre-treatment with KN93 did not alter the increases in eIF2α phosphorylation or levels of ATF4 and CHOP caused by 4,4′-Br_**2**_DIM (Supplementary Fig. S1H). In addition, the expression of the beta and gamma subunits of CaMKII in LNCaP C42B, and DU145 cells was confirmed (Fig. 7E). The alpha and delta subunits of CaMKII were not found (data not shown).

## DISCUSSION

### Ring-DIMs kill AD and AI prostate cancer cells but not non-tumourigenic RWPE-1 cells

We have previously reported that ring-DIMs and DIM induce apoptosis and necrosis in androgen receptor-positive (AR+) AD LNCaP and in androgen receptor-negative (AR-) AI PC-3 prostate cancer cells. Similar to our previous report [17], 4,4′-Br_**2**_DIM induced cell death in AD AR+ LNCaP, AI AR+ C42B and AI AR- DU145 cells with the greatest potency of all five compounds tested. Concentrations of ring-DIMs that killed 100% of prostate cancer cells after 24 hours (20 μM for 4,4′-Br_**2**_DIM, 30 μM for 7,7′-Br_**2**_DIM, 30 μM for 7,7′-Cl_**2**_DIM, 50 μM for 4,4′-Cl_**2**_DIM and 50 μM for DIM) were not toxic to non-tumourigenic RWPE-1 prostate epithelial cells (Fig. 1). This confirms that DIM and its 4,4′- and 7,7′- ring-substituted analogs selectively affect processes and pathways that are dysregulated in cancerous tissues, and are not toxic to normal cells.

### Cell death induced by the ring-DIMs is dependent on mitochondrial dysfunction but not ER stress or autophagy

In our previous study, we hypothesized that 4,4′-Br_**2**_DIM induces cell death by independently activating both intrinsic and extrinsic apoptosis pathways based on the observation that this derivative activated caspases-8 and -9, but did not increase Bid cleavage. We further hypothesized that ER stress plays a role in ring-DIM-induced cell death, as both 4,4′-Br_**2**_DIM and 7,7′-Cl_**2**_DIM induced increased expression of DR4 and DR5, and 4,4′-Br_**2**_DIM also increased Fas receptor and Fas ligand (FasL) [17]. Here, we show that ER stress and mitochondrial dysfunction are early events in ring-DIM- and DIM-induced prostate cancer cell death. ATF4 expression was increased by 4,4′-Br_**2**_DIM within 1 hour of exposure, while 7,7′-Cl_**2**_DIM and DIM increased ATF4 expression after 4 hours. We observed a significant decrease in MMP in cells treated with 4,4′-dihaloDIMs and DIM, but only a slight decrease in mitochondrial activity of cells treated with 7,7′-dihaloDIMs (Fig. 2). CsA, a potent inhibitor of the mitochondrial permeability transition pore (mPTP) complex, completely abrogated ring-DIM-induced death of LNCaP and C42B cells, but could not prevent DIM-induced cell death (Fig. 3). CsA also abrogated the toxicity of 4,4′-Br_**2**_DIM in DU-145 cells (Supplementary Figure S1B, E). The loss of MMP was inhibited by CsA in cells treated with the ring-DIMs as well as DIM, suggesting that mitochondrial dysfunction is necessary for cell death caused by the ring-DIMs but not DIM. However, pre-treatment with CsA did not abrogate eIF2α phosphorylation or cause a decline in the levels of ATF4 or CHOP in cells treated with either 4,4′-Br_**2**_DIM, 7,7′-Cl_**2**_DIM or DIM (Fig. 3E-G). Therefore, our results suggest that ring-DIM-mediated ER stress is activated in parallel, and not downstream, of mitochondrial dysfunction. It has been reported previously that eIF2α could play a role in the survival of tumourigenic cells in response to Akt inhibition [39], and DIM has been shown to be a potent inhibitor of the Akt pathway [15, 40]. Our *in silico* data support the notion that DIM may act as a direct Akt inhibitor, as Akt showed a significant binding affinity to DIM and the ring-DIMs (Supplementary Table S1). Therefore, the observed induction of ER stress may be a pro-survival response to the mitochondrial disruption caused by the ring-DIMs and DIM, and is not necessary for ring-DIM-induced toxicity.

Although ER stress does not appear to be responsible for ring-DIM-induce cell death, we wished to confirm this using salubrinal, a compound commonly used to inhibit ER stress by blocking dephosphorylation of eIF2α. Pre-treatment with 20 μM of salubrinal inhibited cell death caused by 4,4′-Br_**2**_DIM and 4,4′-Cl_**2**_DIM in LNCaP and C42B cells, and by 4,4′-Br_**2**_DIM in DU145 cells. In contrast, pre-treatment with salubrinal sensitized LNCaP and C42B cells to the toxicity of 7,7′-Br_**2**_DIM, 7,7′-Cl_**2**_DIM and DIM (Fig. 4A, B). Interestingly, the increased phosphorylation of eIF2α mediated by 4,4′-Br_**2**_DIM was abrogated by pre-treatment with salubrinal in all three cell lines (Fig. 4E; Supplementary Fig S1G). However, in LNCaP and C42B cells, pre-treatment with salubrinal did not consistently abrogate the 4,4′-Br_**2**_DIM-induced levels of CHOP or ATF4 (Fig. 4E), and in DU145 cells salubrinal only partially reduced the 4,4′-Br_**2**_DIM-induced expression of these markers of ER stress (Supplementary Fig S1G). Moreover, salubrinal alone did not affect phosphorylation of eIF2α in all three cell lines (Fig. S2A, B), suggesting that, in prostate cancer cells, salubrinal does not directly modify the phosphorylation status of eIF2α; nor does it significantly inhibit the onset of ring-DIM-mediated ER stress. These results are consistent with two other reports showing that salubrinal did not modify the phosphorylation status of eIF2α, and interacted directly with Bcl-2 [41, 42]. We propose that the mitochondrion is a novel target of salubrinal which differentially modulates the disruption of mitochondrial stability caused by the ring-DIMs: whereas salubrinal abrogates 4,4′- Br_**2**_DIM-mediated loss of MMP (Fig. 4C, D), it exacerbates the loss of MMP induced by 7,7′-dihaloDIMs and DIM (Fig. 5C, D). Furthermore, it appears that salubrinal's ability to modulate the cell death caused by the ring-DIMs is dependent on the loss of MMP, as pre-treatment with CsA negated the synergistic cytotoxic effects of co-treatment with salubrinal and 7,7′-dihaloDIMs or DIM (Fig. 5A-D). Future studies will investigate the mitochondrial target(s) affected by salubrinal, and how they may be utilized to further increase the cytotoxic potency of the 7,7′-dihaloDIMs and DIM in prostate cancer cells.

DIM has been shown to induce ER stress-dependent autophagy in ovarian cancer cells, with the autophagic response being partially responsible for DIM-mediated cell death *in vitro* and *in vivo* [27]. In contrast, our studies show that DIM- and ring-DIM-mediated autophagy is cytoprotective, since pre-treatment with either bafilomycin A1, 3-MA or transcriptional silencing of LC3B sensitized both LNCaP and C42B cells to cell death induced by DIM or the ring-DIMs. However, in DU145 cells, which are autophagy-deficient due to a mutation in the *ATG5* gene leading to the premature termination of ATG5 transcripts [43], bafilomycin A1 and 3-MA had no effect (Fig. 6). Additionally, Kandala and Srivastava [27] provided evidence that DIM mediated autophagy was dependent on CHOP signalling. However, in LNCaP, C42B and DU145 cells, ER stress was not sufficient to induce cell death in prostate cancer cells. Thus, it is possible that the onset of ER stress might also serve a cytoprotective function in prostate cancer cells by activating autophagy via CHOP signaling.

### Is Ca^2+^/calmodulin-dependent protein kinase (CaMKII) involved in 4,4′-Br_2_DIM-mediated mitochondrial dysfunction?

Our *in silico* docking experiments revealed CaMKII as a potential high affinity target for the ring-DIMs and DIM (Table 1). In prostate cancer cells, CaMKII activity has been implicated in cell survival and loss of androgen-dependence [44]. However, in a variety of cases, CaMKII can be pro-apoptotic and in hepatocellular carcinoma cells CaMKII is necessary for melittin-induced apoptosis [13], and CaMKII activation has been linked to sustained c-Jun N-terminal kinase and p38 mitogen-activated kinase stress signaling [13, 45, 46]. Moreover, CaMKII has been shown to play a role in the simultaneous induction of ER stress and mitochondrial dysfunction [46]. We observed that KN93, a selective inhibitor of CaMKII, but not its inactive form KN92, prevented the death of cells treated with 4,4′-Br_**2**_DIM, but not 7,7′-Cl_**2**_DIM or DIM (Fig. 7A, B). In addition, KN93 abrogated the loss of MMP observed after exposure to 4,4′-Br_**2**_DIM and decreased eIF2α phosphorylation, but did not significantly prevent expression of ATF4 or CHOP (Fig. 7D). Thus, CaMKII activation may directly initiate the intrinsic pathway of apoptosis in prostate cancer cells, and this effect is independent of induction of ER stress. Interestingly, we found protein expression of only the beta and gamma subunits of CaMKII in the three prostate cancer cell lines tested (Fig. 7E), with both subunits exhibiting high docking affinity values for 4,4′-Br_**2**_DIM (Table 1). The crystal structure of CaMKII-beta and the docking site of each ring-DIM and DIM are shown in Supplementary Fig. S3. C42B cells have been shown to transcriptionally express all four subunits of CaMKII (alpha, beta, gamma, delta), whereas LNCaP and DU145 cells only expressed the beta, gamma and delta transcripts [47]. Our study confirms that the CaMKII-beta and -gamma proteins are indeed produced from these transcripts, but that protein levels of the CaMKII-alpha and -delta subunits are undetectable in each of the three cell lines. These data suggest that CaMKII may be a selective upstream target of 4,4′-Br_**2**_DIM that is responsible for the early onset of mitochondrial dysfunction and ER stress in prostate cancer cells. Although our *in silico* analysis identified a potential interaction between CaMKII and 7,7′-Cl_**2**_DIM and between CaMKII and DIM, it appears that CaMKII activity is not necessary for 7,7′-Cl_**2**_DIM- or DIM-mediated cell death. This differential response between the 4,4′-dihalo- and 7,7′-dihaloDIMs to inhibition of CaMKII by KN93 provides further evidence that the mechanisms of action of the ring-DIMs is highly structure-dependent. We previously showed that the 4,4′-dihaloDIMs, but not the 7,7′-dihaloDIMs induced the Fas receptor and FasL in LNCaP cells [17]; additionally, the 7,7′-dihaloDIMs were shown to be more potent inhibitors of DHT-mediated LNCaP cell proliferation than the 4,4′-dihalo-DIMs [17]. In the present study we also show that the 4,4′- and 7,7′-dihaloDIMs elicit opposite responses in prostate cancer cells pre-treated with salubrinal, but that these effects are unrelated to ER stress. Moreover, pre-treatment of all three cell lines with KN93 did not prevent the increase in expression of either CHOP or ATF4, further confirming that ER stress is not a key contributor to 4,4′-Br_**2**_DIM-mediated cell death.

## CONCLUSION

**Figure 8:**
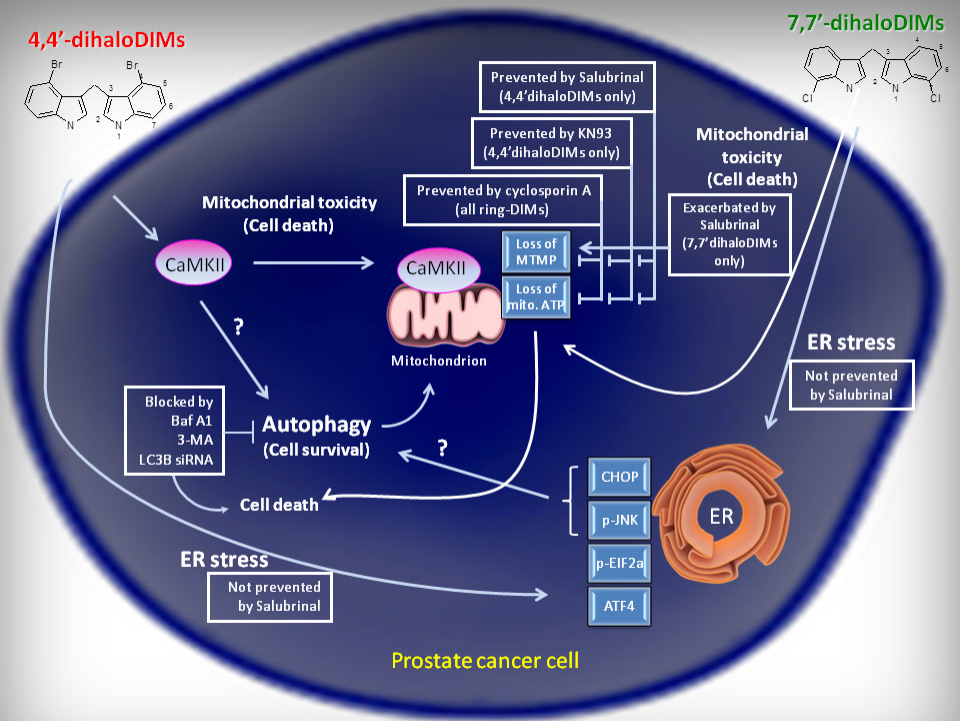
A schematic representation of the differential pathways activated by the 4,4′- and 7,7′-dihalogenated ring-DIMs and their role in ring-DIM-induced prostate cancer cell death

We have confirmed that ring-substituted dihaloDIMs act via distinct, structure-dependent, yet overlapping mechanisms (Figure 8) to induce potent cytotoxic effects in AD and AI prostate cancer cells, but not normal prostate epithelium. We have shown that cell death mediated by the most potent ring-DIM, 4,4′-Br_**2**_DIM, is dependent on CaMKII activation and subsequent mitochondrial dysfunction, and that ER stress is insufficient to induce cell death in response to either the ring-DIMs or DIM. We also show that the ring-DIMs and DIM induce protective autophagy in prostate cancer cells. Future studies will concentrate on the relationship between 4,4′-Br_**2**_DIM, CaMKII and mitochondrial dysfunction, the effectiveness of this potent anti-cancer compound in animal models of prostate cancer, and the potential for targeting the ER stress and autophagy pathways in order to increase the effectiveness of the ring-DIMs and DIM as anti-neoplastic agents.

## MATERIALS AND METHODS

### Cell lines and reagents

LNCaP, and DU145 human prostate cancer cells as well as RWPE-1 immortalized normal prostate epithelial cells were purchased from the American Type Culture Collection (Manassas, VA). LNCaP C4-2B (C42B) cells were purchased from the MD Anderson Cancer Center (Houston, TX). LNCaP, C42B, DU145, and RWPE-1 cells were grown in RPMI 1640 supplemented with 10% fetal bovine serum, 2 mM L-glutamine, 1% HEPES, 1% sodium-pyruvate and 10ml/L of 100x antibiotic-antimycotic solution (Sigma-Aldrich, St-Louis, MO). Cells were maintained in a humidified atmosphere (5% CO_**2**_) at 37_o_C. Ring-substituted 4,4′- and 7,7′-dihaloDIMs were synthesized in our laboratories at >95% purity and were dissolved in 100% dimethyl sulfoxide (DMSO) to obtain 100 mM stock solutions. Dihydrotestosterone (DHT; Steraloids Inc., Newport, RI) was dissolved in DMSO to make a 100 mM stock solution. Cyclosporin A (CsA; Cell Signaling, Beverly, MA), salubrinal (Enzo Life Sciences, Farmingdale, NY), KN92, KN93 (Millipore, Billerica, MA) and bafilomycin A1 (Sigma Aldrich) were dissolved in DMSO as 1000-fold concentrated stock solutions. The final concentration of DMSO in culture medium was 0.1% for single exposures and not greater than 0.3% for combined exposures.

### Treatment of cells with ring-DIMs, pharmacological inhibitors and siRNA

LNCaP, C42B, and DU145 cells were exposed to the ring-DIMs at the indicated final concentrations in their respective culture medium, supplemented with 2% dextran-coated charcoal-stripped FBS. DHT was added to LNCaP cells after serial dilution in DMSO to a working stock solution of 100 nM, resulting in a final DHT concentration of 0.1 nM in culture medium. CsA, KN92 and KN93, salubrinal, bafilomycin A1 were added to cell cultures 4 hours prior to treatment with either ring-DIMs, DIM or DMSO vehicle control. 3-Methyladenine was dissolved directly in water and added freshly to the cells 4 hours prior to exposure to the diindolylmethane compounds.

For siRNA experiments, LNCaP or C42B cells were transfected with SMARTpool *ON-TARGETplus* siRNA oligonucleotide for *LC3B* (Dharmacon, USA) using lipofectamine RNAiMAX (Life Technologies, USA) in serum free Opti-MEM according to manufacturer's protocols. ON-TARGETplus Non-targeting Control siRNAs was used a negative control. After a 24-hour incubation, transfected cells were exposed to the ring DIMs, DIM or vehicle control for a further 24 hours.

### Cell death and mitochondrial membrane potential (MMP)

For cell death measurements, LNCaP, C42B, and DU145 cells were seeded in 24-well plates in 2% stripped FBS. Cells were then treated with (LNCaP) or without (C42B, DU145) 0.1 nM DHT and several concentrations of ring-DIMs, DIM or vehicle control (DMSO). After 24 hours, Hoechst 33342 (Sigma-Aldrich) and propidium iodide (PI; Invitrogen, Carlsbad, CA) stains were both added to each well at a concentration of 1 μg/ml (in water) after which the plates were incubated for 15 minutes at 37_o_C. Hoechst- and PI-positive cells were then counted under a Nikon Eclipse (TE-2000U) inverted fluorescence microscope at 20x magnification using filter cubes with excitation wavelengths of 330-380 and 532-587 nm, respectively. Intact cells were counted as exhibiting neither chromatin condensation, chromatin fragmentation nor PI staining. To measure MMP, tetramethylrhodamine ethyl ester (TMRE) was added to each well at a final concentration of 50 nM for 15 minutes at 37_o_C. TMRE is a cell permeable, positively charged dye that accumulates in active negatively charged mitochondria. In inactive or (partially) depolarized mitochondria, membranes have decreased potential and fail to sequester TMRE. Cells were then observed under an inverted fluorescence microscope using a filter cube with an excitation wavelength of 532-587 nm. The photos of cells treated with either TMRE, Hoechst 33342 or PI were analyzed using ImageJ image processing software [29].

### ATP measurements

LNCaP, C42B, and DU145 cells were seeded in 96-well plates in 2% stripped-FBS. Cells were then treated with (LNCaP) or without (C42B, DU145) 0.1 nM DHT and several concentrations of ring-DIMs, DIM or DMSO alone. A 0.5 M stock solution of 2-deoxy-D-glucose (Sigma-Aldrich) was prepared in water and added to the wells at a final concentration of 5 mM 4 hours prior to addition of ring-DIMs, DIM or DMSO alone. Mitochondrial ATP levels were measured 1 hour later using a ViaLight Plus kit (Lonza, Basel, Switzerland). Briefly, cells were treated for 10 minutes with lysis buffer, after which a bioluminescent ATP monitoring reagent was added to each well for 2 minutes. The bioluminescent signal was measured using a SpectroMax M5 microplate reader (Molecular Devices, Sunnydale, CA).

### SDS-PAGE and immunoblotting

Crude protein extracts (50 μg) were resolved by electrophoresis in 10% sodium dodecyl sulfate-polyacrylamide gels and then transferred to PVDF Immobilon-P membranes (Bio-Rad, Mississauga, ON). Blots were blocked using 5% milk powder (Selection brand, Marché Jean-Talon, Montréal, QC) and incubated with antibodies using a dilution of 1:500 for anti-β-actin, a 1:250 dilution for anti-CaMKII beta subunit and anti-CaMKII gamma subunit (Santa Cruz Biotechnology, Santa Cruz, CA), a 1:500 dilution for anti-CHOP, a 1:1000 dilution for anti-LC3B, anti-ATF4, anti-phospho eIF2α (Ser51), and a 1:10000 dilution for anti-eIF2α (Cell Signaling). Immunoreactive proteins were exposed to anti-rabbit or anti-mouse horseradish peroxidise-conjugated secondary antibodies (Millipore) that were diluted 1:5000. Antigen-antibody complexes were detected using Immobilon ECL Western Chemiluminescent HRP Substrate (Millipore) and recorded with a VersaDoc imaging system (Bio-Rad).

### *In Silico* 3-D affinity docking analyses

The structures of DIM and the ring-DIMs were optimized in a Gaussian 09 program package (Frisch et al., 2004) by the Density Functional Theory (DFT) method at the B3LYP/6-31G level. The output file was translated to pdb and pdbqt formats using Open Babel [30] and AutoDock Tools [31], respectively. The 3-D structures of the proteins were downloaded from the Protein Data Bank (PDB) in pdb format [32]. The structure of TrailR1 was obtained from ModBase because its crystal structure was not reported in PDB. Protein structures were then prepared using Sybyl-X 2.0 (Tripos, St. Louis, MO). In this process, all ions, water molecules and other substructures were removed [33]. We also fixed all side-chains, backbones and protonation types. Once prepared, proteins underwent a two-step optimization procedure using Sybyl-X 2.0. The first step included the Powell method applying Kollman”s united force field AMBER (Assisted Model Building with Energy Refinement) charges, dielectric constant 1.0, NB cutoff 8.0, maximum interactions 1000 and termination gradient 0.001 kcal/mol. The second step utilized Kollman's All Atom approach with the same parameters. The resultant structures were saved as pdb files and then converted to pdbqt format using AutoDock Tools [31]. Kollman charges and polar hydrogen atoms were added to the 3-D structures of the proteins using the same software [34].

Docking and docking-refinement experiments were carried out through a blind docking strategy by AutoDock Vina [35] to allow inclusion of the whole protein surface and all possible binding sites. The 3-D docking grid was centered on the macromolecule [36] and coordinates were calculated with a resolution of 0.357 Å using AutoDock Tools [31]. Docking analyses were performed with the AutoDock Vina 1.1 molecular docking and virtual screening program [35] running on a Linux operating system using the following settings: energy range = 1.5, number of modes = 20 and exhaustiveness = 25. The docking models of protein/DIM and protein/ring-DIM complexes with high-affinity scores (less than −8.0 kcal/mol) were refined with repetitions of 100 runs to increase accuracy and identify alternative *in silico* binding sites for each compound.

Protein/compound complexes underwent conformational analyses to determine the contact residues and interaction type using LigandScout 3.1 software with default settings [37]. The interaction cutoff threshold of the pdb interpretation, which defines a sphere around the ligand, was set at 7.0 Å. The atoms of the protein found inside the ligand-sphere were considered to be able to interact with the ligand [38].

### Statistical analyses

All experiments were performed in at least triplicate and results presented as mean ± SEM. Statistically significant differences (* P < 0.05, ** P < 0.01, *** P < 0.005) were calculated using a two-tailed Student t-test. IC_**50**_ values were calculated using nonlinear curve-fit analysis. All analyses were performed using GraphPad Prism v5.03 (GraphPad Software, San Diego, CA).

## SUPPLEMENTARY MATERIAL AND FIGURES


