# Erratum: 3,3′-Diindolylmethane (DIM) and its ring-substituted halogenated analogs (ring-DIMs) induce differential mechanisms of survival and death in androgen-dependent and–independent prostate cancer cells

**DOI:** 10.18632/genesandcancer.98

**Published:** 2016-01

**Authors:** Alexander A. Goldberg, Hossam Draz, Diana Montes-Grajales, Jesus Olivero-Verbél, Stephen H. Safe, J. Thomas Sanderson

Genes Cancer. 2015 May; 6(5-6): 265-280.

PMCID: PMC4482247 PMID: 26124925

DOI: 10.18632/genesandcancer.60

The acknowledgements section is missing in the publication.

**ACKNOWLEDGEMENTS**

This study was supported financially by a grant from the Canadian Institutes of Health Research (CIHR, grant number MOP 115019).

