# Supplementary material for: 3,3′-Diindolylmethane (DIM) and its ring-substituted halogenated analogs (ring-DIMs) induce differential mechanisms of survival and death in androgen-dependent and –independent prostate cancer cells
Source: Genes Cancer. 2015 May;6(5-6):265–80. doi: 10.18632/genesandcancer.60 (PMC4482247; doi:10.18632/genesandcancer.60)
Supplement: Supplementary file 1 [file ganc-06-265-s001.pdf]

3,3'-Diindolylmethane (DIM) and its ring-substituted halogenated analogs (ring-DIMs) induce differential mechanisms of survival and death in androgen-dependent and –independent prostate cancer cells

Supplementary Material

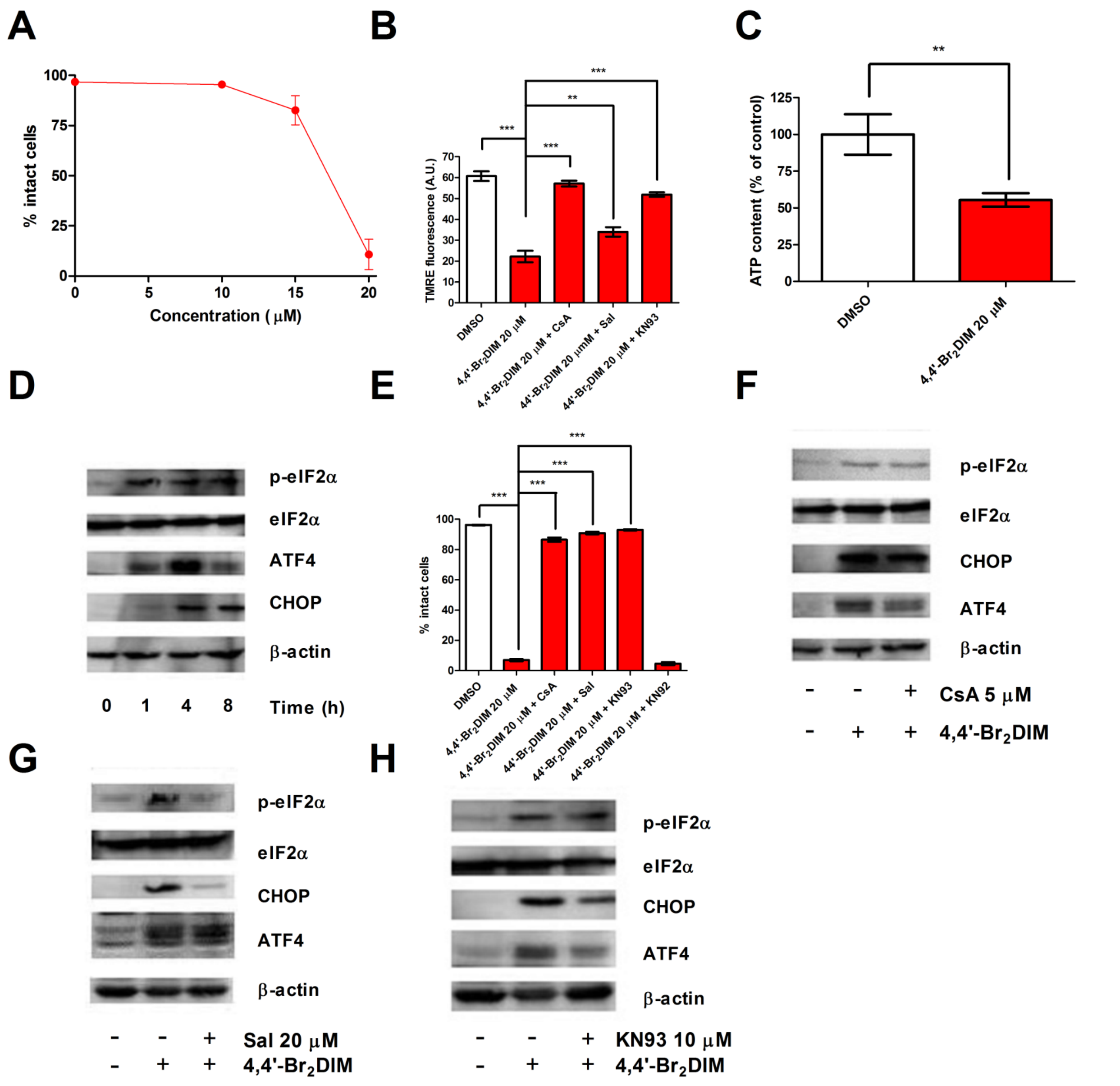

Supplementary Figure S1: Effects of 4,4'-Br$_2$DIM on the AI prostate cancer cell line DU145. (A) Percentage of intact DU145 cells treated with increasing concentrations (5-20 μM) of 4,4'-Br$_2$DIM. (B) TMRE fluorescence of DU145 cells after a 4 hour exposure to 4,4'-Br$_2$DIM with or

without a 4 hour pre-treatment with either CsA, Sal or KN93. (C) Relative mitochondrial ATP levels of DU145 cells treated with 5 mM 2-deoxy-D-glucose after a 4 hour exposure to 4,4'-Br_2DIM with or without a 4 hour pre-treatment with either CsA, Sal or KN93. (D) Phosphorylation of eIF2α, and levels of ER stress proteins were assayed by immunoblot of DU145 cells after 0, 1, 4 and 8 hours of exposure to 4,4'-Br_2DIM. (E) Percentage of intact DU145 cells after a 24 hour exposure to 4,4'-Br_2DIM, with or without a 4 hour pre-treatment with either CsA, Sal, KN92 or KN93. Phosphorylation of eIF2α, and levels of ER stress proteins were assayed by immunoblot of DU145 cells after 24 hrs of exposure to 4,4'-Br_2DIM with or without a 4 h pre-treatment with either CsA (F), Sal (G) or KN93 (H).

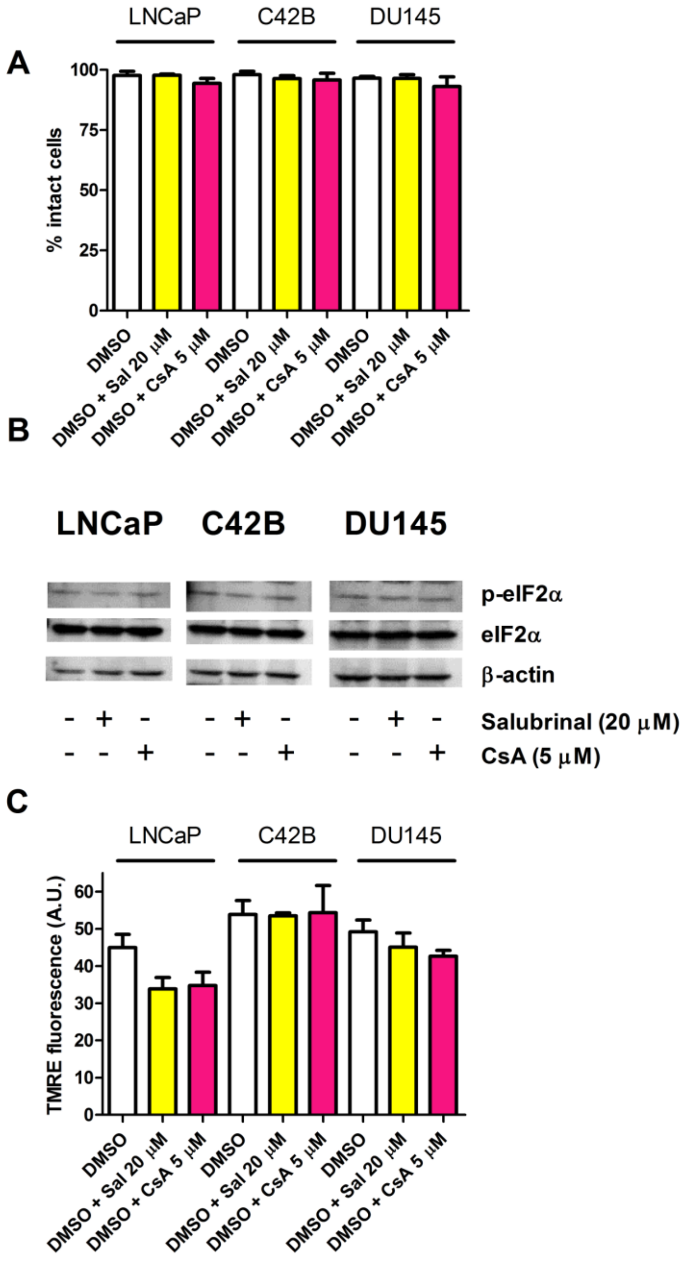

Supplementary Figure S2: Neither salubrinal nor CsA alone influences eIF2α phosphorylation. (A) Percentage of intact LNCaP, C42B, or DU145 cells exposed to either salubrinal or CsA for 24 hours. (B) Phosphorylation of eIF2α in LNCaP, C42B, and DU145 cells exposed to salubrinal or CsA for 24 hours. (C) TMRE fluorescence of LNCaP, C42B or DU145 cells exposed to salubrinal or CsA for 24 hours.

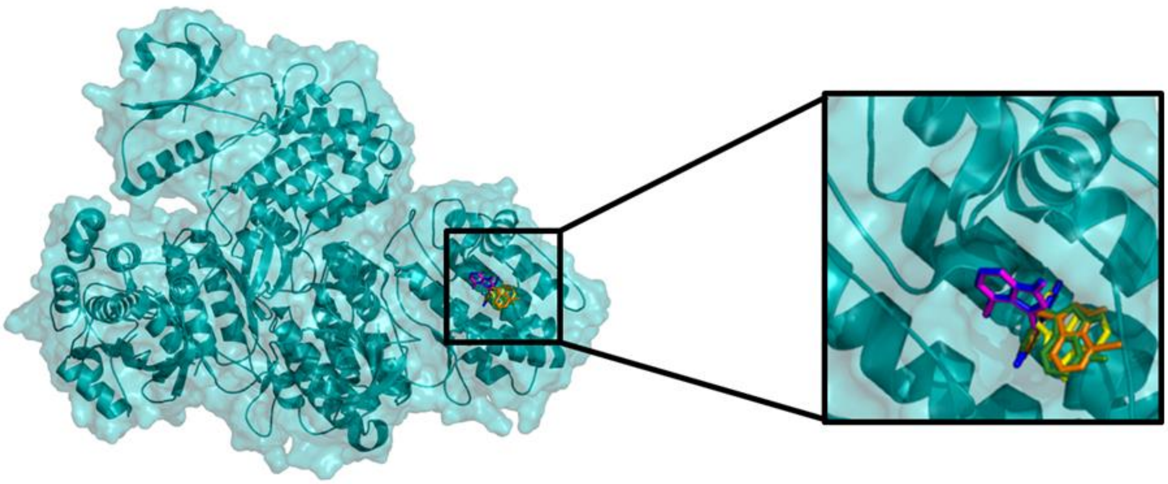

Supplementary Figure S3: Three-dimensional view of the docking between CaMK-II subunit beta (protein database: 3BHH) and diindolylmethane (DIM; yellow), and its derivatives 4,4'-dibromoDIM (blue); 4,4'-dichloroDIM (magenta); 7,7'-dibromoDIM (orange) and 7,7'-dichloroDIM (green).
